# Hybrid endoscopic rescue following endoscopic ultrasound-guided choledochoduodenostomy

**DOI:** 10.1055/a-2774-3396

**Published:** 2026-02-24

**Authors:** Miguel Martins, Joana Mota, Filipe Vilas-Boas, Joel Ferreira-Silva, Eduardo Rodrigues-Pinto, Guilherme Macedo

**Affiliations:** 1 679497 Gastroenterology Department, São João Local Health Unit, Porto, Portugal; 2 26705 University of Porto, Faculty of Medicine, Porto, Portugal

**Fig. 1:**
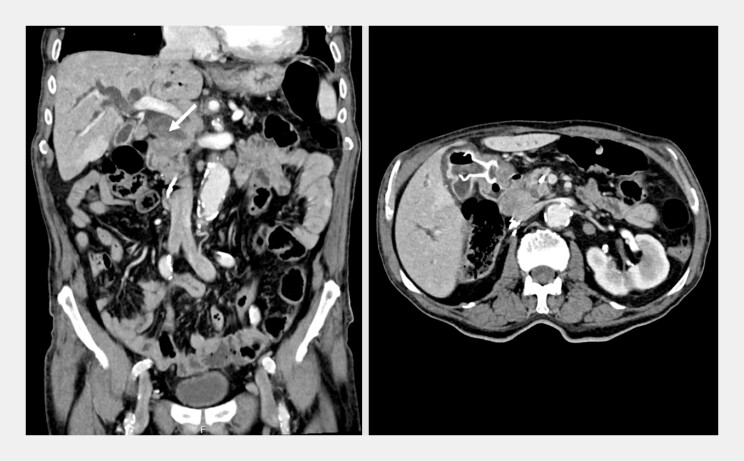
A CT scan showing biliary obstruction caused by a mass in the pancreatic head, with a previously placed cholecystogastrostomy lumen-apposing metal stent (LAMS) in situ. CT, computed tomography.

**Fig. 2:**
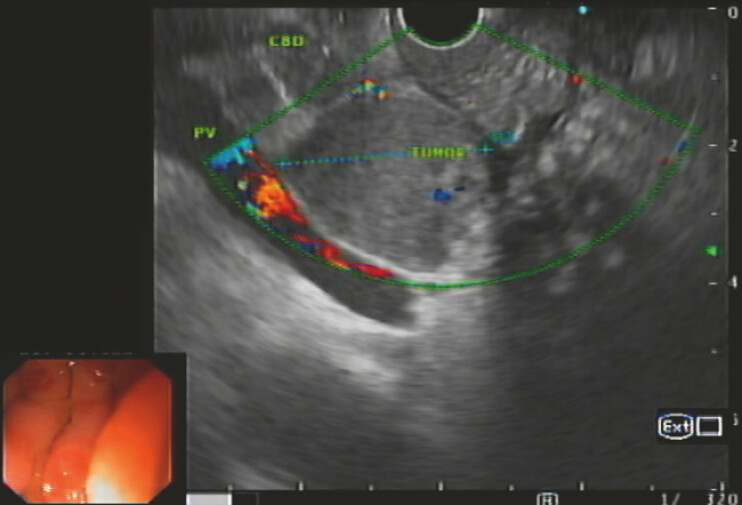
An endoscopic ultrasound image showing common bile duct (CBD) dilation caused by the pancreatic head lesion.

An 88-year-old man with biliary obstruction caused by a complex cystic lesion in the pancreatic head, considered inoperable due to significant comorbidities, was referred for endoscopic biliary drainage. Following failed endoscopic retrograde cholangiopancreatography, endoscopic ultrasound (EUS)-guided cholecystogastrostomy was performed, as the cystic duct was patent. Two years later, he presented with cholangitis from recurrent biliary obstruction due to disease progression ( Fig. 1 ). EUS revealed common bile duct (CBD) dilation (~14 mm), caused by the pancreatic head lesion ( Fig. 2 ). Left intrahepatic bile duct dilation was minimal, precluding hepaticogastrostomy. Decision was to perform EUS-guided choledochoduodenostomy.

**Fig. 3:**
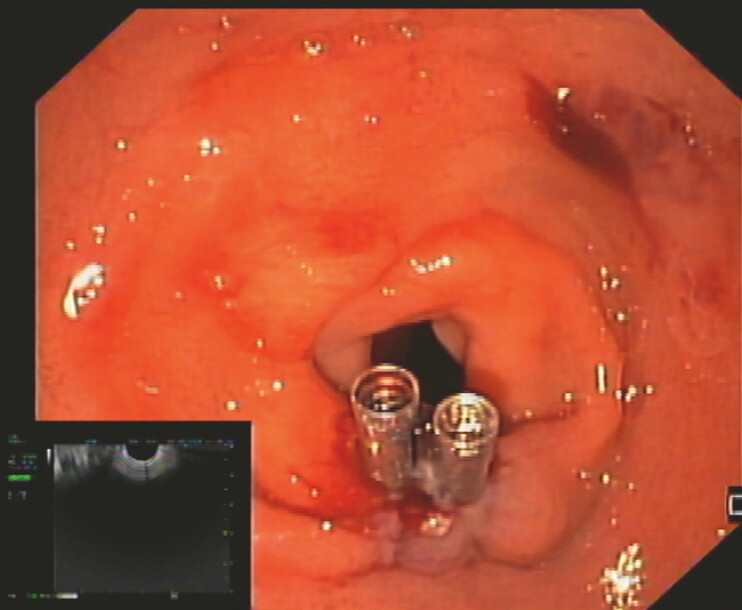
An endoscopic view of the duodenal bulb showing the closure of the wall defect resulting from the initial drainage attempt complicated by type 1 lumen-apposing metal stent (LAMS) misdeployment.

**Fig. 4:**
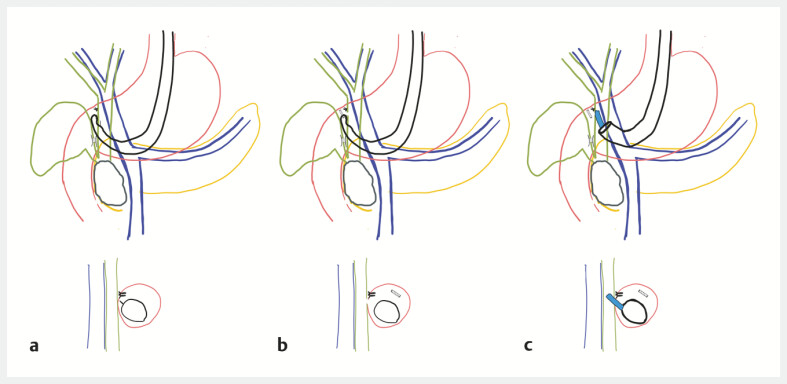
Schematic representation of procedural adverse events and their management: **a** Duodenal wall defect closure after lumen-apposing metal stent (LAMS) misdeployment. **b** Stent migration during the second attempt, resulting in biliary and duodenal defects. **c** The hybrid endoscopic rescue procedure using cholangioscopy for recannulation of the biliary fistula and stent repositioning.

**Fig. 5:**
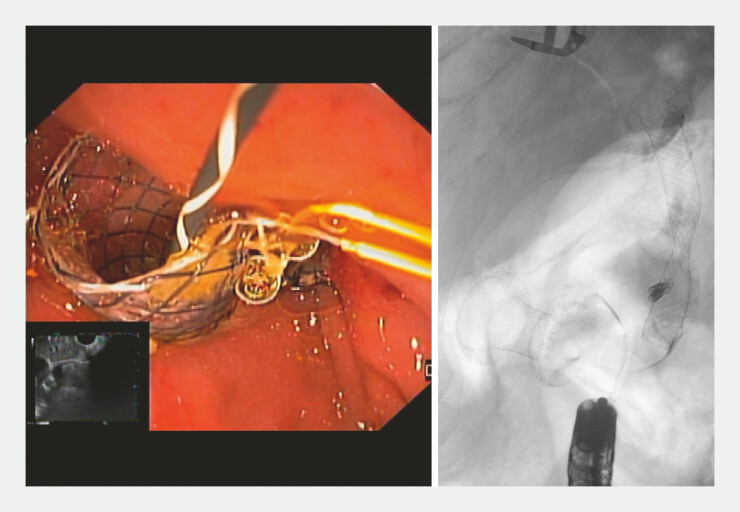
Final endoscopic and fluoroscopic images demonstrating successful biliary drainage.

The first attempt with a 6 × 8 mm electrocautery-tip lumen-apposing metal stent (Axios, Boston Scientific) using the free-hand technique resulted in a type I misdeployment 1 . The stent was removed, and the duodenal wall defect was closed with two through-the-scope clips ( Fig. 3 ). The second attempt involved CBD puncture using a 19G needle (Expect Slimline, Boston Scientific) for cholangiography and duct distension, fistula creation using an 8.5 Fr cystotome (Cysto-Gastro-Set, Endo-Flex), and the placement of the 10 × 60 mm fully covered metal biliary stent (Evolution, Cook Medical); however, the stent migrated distally during deployment ( Fig. 4 ). Despite multiple attempts, CBD re-canulation using guidewire manipulation was not achieved. Using a therapeutic endoscope (Olympus GIF-2TH180), a cholangioscope (SpyGlass DS II, Boston Scientific) was advanced through the fistula into retroperitoneal space, enabling access to the bile duct and stent placement over a guidewire ( Video 1 ). Final endoscopic and fluoroscopic imaging confirmed pneumobilia and adequate bile/contrast drainage ( Fig. 5 ). Patient recovered rapidly, being discharged on post-procedure day 3.

Cholangioscopy-assisted recannulation of the biliary fistula after stent migration, enabling guidewire reposition and deployment of a fully covered metal stent to restore effective biliary drainage.Video 1

EUS-guided biliary drainage is an effective alternative when transpapillary drainage is not feasible, although adverse events can be significant 2 . To our knowledge, the use of cholangioscopy for biliary fistula salvage after EUS-choledochoduodenostomy has not been previously reported 3 . This hybrid NOTES-based approach may represent a valuable rescue option in such cases.

Endoscopy_UCTN_Code_CPL_1AL_2AD
